# Antimicrobial activity of different Lactobacillus species against multi- drug resistant clinical isolates of *Pseudomonas aeruginosa*

**Published:** 2011-03

**Authors:** H Jamalifar, HR Rahimi, N Samadi, AR Shahverdi, Z Sharifian, F Hosseini, H Eslahi, MR Fazeli

**Affiliations:** 1 Departments of Drug and Food Control; 2 Biotechnology; 3 Toxicology and Pharmacology, Faculty of Pharmacy, Tehran University of Medical Sciences, Tehran, Iran; 4 Department of Drug Regulatory Affairs, Iranian Food and Drug Administration, Tehran, Iran; 5 Pharmaceutical Quality Assurance Research Center, Tehran, Iran.

**Keywords:** Lactobacillus species, Anti-pseudomonal activity, *Pseudomonas aeruginosa*

## Abstract

**Background:**

Lactobacilli are the well known friendly bacteria for their probiotic activities against pathogens. The inhibitory activity of different strains of lactobacilli either obtained as commercial products or isolated from human feces was investigated against the clinical isolates of *Pseudomonas aeruginosa*. The isolates were selected as the most resistant strains when challenged with anti-pseudomonal antibiotics already in clinical practice.

**Materials and Methods:**

Both the plate spot test as well as the agar cup method were used for screening of *Lactobacillus* strains against *Pseudomonas aeruginosa*.

**Results:**

A *Lactobacillus acidophilus* strain isolated from feces of an Iranian child showed a strong anti-pseudomonal activity (90 percent after 72h incubation) against the multi-drug resistant clinical isolates while a *Lactobacillus reuteri* strain isolated from a commercial oral product resulted in relatively weak response and a *Lactobacillus acidophilus* strain isolated from a commercial vaginal product did not show any inhibitory activity. In a kinetic study the lactobacillus sensitive *Pseudomonas aeruginosa* showed a significant bacteriostatic activity in vitro in the presence of lactobacillus supernatants.

**Conclusion:**

Some lactobacilli exhibit significant inhibitory activity against the multidrug resistant clinical isolates of *Pseudomonas aeruginosa*.

## INTRODUCTION

Multi-drug resistant bacteria are the cause of numerous clinical problems throughout the world. Increased resistance among pathogens causing nosocomial and community acquired infections is known to be related to the widespread utilization of antibiotics (1). Infectious diseases caused by resistant microorganisms are accountable for increased health costs as well as high morbidity and mortality, especially in developing countries. *Pseudomonas aeruginosa* is an opportunistic gram negative bacterium which is a major cause of nosocomial infections, usually occurring in the context of serious underlying diseases and accounting for nearly 10% of all hospital-acquired infections of surgical sites, the respiratory tract and the urinary tract (2, 3). It is also prevalently related to otitis media and nasal infections and represents a leading cause of morbidity due to burn wound infection (4, 5). *P. aeruginosa* has inherent resistance to most available antibiotics, including aminoglycosides, anti-pseudomonal penicillins, newer cephalosporins, imipenem and flouroquinolones as treatment options for systemic infections (6–8). Recent reports have documented the role of exogenous Lactobacilli in the prevention and treatment of some infections. Lactobacillus strains are commensal in the human body. Oral administration of Lactobacillus strains has been found to be useful in various bacterial infections (9–11). Its beneficial effect may be associated to its ability to inhibit the growth of pathogens, apparently by the secretion of antibacterial substances including lactic acid, hydrogen peroxide and etc. (12). We undertook this in vitro study to evaluate the effects of different Lactobacilli-either obtained as commercial products or isolated from an Iranian villager child feces and their metabolites on multiple drug resistant clinical isolates of *Pseudomonas aeruginosa*.

## MATERIALS AND METHODS

**Bacterial stains and growth conditions**. A number of 55 clinical isolates of *P. aeruginosa* were collected from Imam and Shariati University Hospitals, Tehran, Iran. Identification of the isolates was conducted using conventional isolation methods (13). *Lactobacillus acidophilus* JFSH was originally isolated from a villager child's stool. *Lactobacillus acidophilus* and *Lactobacillus reuteri* strains were originally isolated from commercial products (14). Lactobacillus strains were grown in Lactobacillus MRS Broth at 37°C for 24 h.

**Susceptibility testing**. Susceptibility of the strains to 12 antibiotics including cephradin, ampicillin, ceftriaxon, chloramphenicol, cefotaxime, ceftazidime, tobramycin, piperacillin, imipenem, gentamicin and amikacin (purchased from Padtan Teb Company, Tehran, Iran) was investigated using Kirby-Bauer disk diffusion method and by comparing their growth inhibition zones to those reported by CLSI (15–18). The diameters of inhibition zones were measured and compared with the zones suggested by CLSI, using susceptible strains as control. From these isolates, three strains were selected for supernatant mixed culture test.

**Antimicrobial assay**. The inhibitory activity of different Lactobacillus strains was screened against multiple drug resistant *Pseudomonas aeruginosa* using conventional agar spot test (19). Furthermore, the effect of supernatants of screened Lactobacillus strains on growth of the *Pseudomonas aeruginosa* was confirmed by the agar cup method (20). Briefly, 24-hr-old positive Lactobacillus cultures in MRS broth were centrifuged at 5500 g for 10 min at 4oC. The supernatants were discarded, and 0.1 ml of the precipitant was used for the study of antibacterial activity. The antibacterial activity of metabolites produced by screened samples of Lactobacillus strains was further investigated by supernatant mixed culture technique. Potent strains were incubated in 5 ml of MRS broth (pH 6.4) and *Pseudomonas spp*. were incubated in 5 ml of Muller-Hinton broth (Ph 7.2), both for 24 hrs in conventional conditions. 1 ml por-tions of supernatants of the centrifuged Lactobacillus cultures were mixed with 1 ml of the test strains cultures (4 × 105 CFU/ml) in Muller- Hinton broth. The optical densities of culture media were measured at 0, 6, 12, 18 and 24 hrs after incubation at 580 nm. Also the CFU were counted by the spread-plate technique.

**Effect of lactic acid, hydrogen peroxide, and buffer on** ***Pseudomonas*** **strains**. The effects of lactic acid (pH 2.0) and hydrogen peroxide (pH 6.5) at the concentrations of 3% v/v on *Pseudomonas spp*. growth were tested by the agar cup method.

## RESULTS

**Fig. 1:**
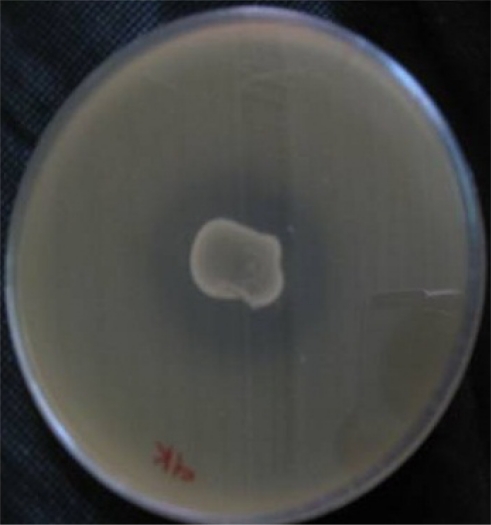
Inhibition effect of *Lactobacillus plantarium* culture supernatant by the agar well diffusion assay.

**Fig. 2:**
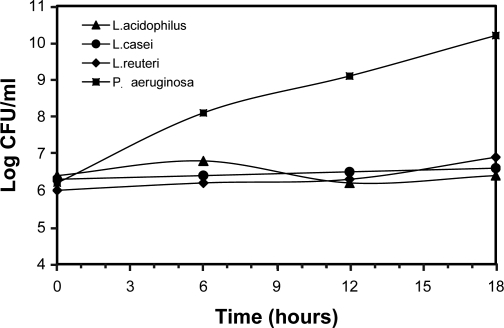
Antibacterial activity of *Lactobacillus casei*, *Lactobacillus acidophilus* and *Lactobacillus reuteri* against *Pseudomonas aeruginosa*. The surviving CFU/ml was quantitated after 0 h, 3 h, 6 h, 9 h, 12 h, 15 h, and 18 h.

The resistant pattern of 55 *P. aeruginosa* to antimicrobial agents is shown in Table 1. Moreover, all tested strains showed resistance against cephradin and ampicillin. Low susceptibility of tested strains against other potent antimicrobial agents is also shown (Table 1). Five potent lactobacillus strains were selected among 200 samples by agar spot test and agar cup method (Fig. 1). These strains were further identified by conventional techniques for characterization of Lactobacillus species (listed in Table 2). The antibacterial activity of the supernatants of these autochthonus Lactobacilli and also commercially *Lactobacillus acidophilus* and *Lactobacillus reuteri* were tested against 10 highly resistant strains of *P. aeruginosa* (Table 2). A *Lactobacillus acidophilus* strain isolated from the feces of an Iranian villager child showed a strong and unchanged activity for 72 hrs against the multiple drug resistant clinical isolates while *Lactobacillus reuteri* and *Lactobacillus gasseri* from an oral product resulted in relatively weak response and a *Lactobacillus acidophilus* strain isolated from a commercial vaginal product did not show any inhibitory activities. Examination of the killing kinetics (Fig. 2) revealed that *Lactobacillus casei*, *Lactobacillus acidophilus* and *Lactobacillus reuteri* had an obvious improvement in killing *P. aeruginosa*. A sensitive *P. aeruginosa* to all Lactobacillus strains was selected as the test strain for time killed curve study.

**Table 1:** Resistance percentage of *P. aeruginosa* to various antimicrobial agents (Total population=55).

**Antibiotics**	*Pseudomonas aeruginosa*	

	Resistant n (%)	Susceptible n (%)
Gentamicin (10 µg)	27 (49.0)	15 (27.2)
Ciprofloxacin (5 µg)	14 (25.4)	32 (58.1)
Amilcacin (30 µg)	24 (43.6)	22 (40.0)
Tobramycin (10 µg)	10 (33.3)	7 (23.3)
Ceftazidime (30 µg)	37 (67.2)	11 (20.0)
Cefotaxime (30 µg)	30 (54.5)	9 (16.3)
Cephradin (30 µg)	55 (100)	0 (0)
Chloramphenicol (30 µg)	9 (16.3)	6 (10.9)
Piperacillin (100 mcg)	42 (76.3)	13 (23.7)
Imipenem (10 mcg)	41 (74.5)	14 (25.5)
Ceftriaxon (30 mcg)	46 (83.6)	9 (16.4)
Ampicillin (30 mcg)	55 (100)	0 (0)

**Table 2:** Inhibitory activity of the supernatants of some Lactobacillus cultures against multiple resistant *P. aeruginosa* at 48 and 72 hrs.

Bacteria	The percentage of inhibitory activity	

	48 (h)	72 (h)
*Lactobacillus plantarium*	40	80
*Lactobacillus acidophilus*	40	90
*Lactobacillus casei*	10	90
*Lactobacillus fermentum*	60	20
*Lactobacillus reuteri*	20	70
*Lactobacillus gasseri*	30	70
*Lactobacillus acidophilus* (commercial)	0	0
H_2_O_2_3%	0	100
Lactic acid 3%	0	100

## DISCUSSION

The inhibitory activity of lactic acid bacteria against some resistant clinical isolates of *P. aeruginosa* has been reported. None of the antimicrobial agents was effective against all the multi-drug tested strains demonstrating the current problem in the treatment of multi-drug resistant nosocomial infections. In previous studies *P. aeruginosa* isolates showed intermediate or fully resistance to antimicrobial agents (21–26). Unfortunately, *P. aeruginosa* strains showed complete resistance against cephradin and ampicillin. Further- more, its susceptibility to other potent antimicrobial agents, including ceftriaxon, chloramphenicol, cefotaxime, ceftazidime, tobramycin, piperaciliin, imipenem, gentamicin and amikacin,which are used more in hospital infections, was also tested. Lactic acid bacteria are dispersed in nature such as in dairy, fish, vegetable and grains. They are also found in normal vaginal flora and protect the vagina from urinary tract infection. In fact, many strains of the genus lactobacillus are capable of colonizing specific parts of the body, e.g. the oral cavity and the gastrointestinal and uro-genital tract, where they play an important role in the competitive exclusion of pathogen (27, 28). Antimicrobial activity of *Lactobacillus* strains against bacterial pathogens emerges to be multifactorial and to include the production of hydrogen peroxide, lactic acid, bacteriocin-like molecules and unknown heat-stable, non-lactic acid molecules (29). Other mechanisms proposed for their activity are competition for nutrients (30, 31), and adhesion inhibition of pathogens to surface and simulation of the immune system (32). The results show that the metabolites of lactoba-cillus acidophilus and also two other strains could suppress the growth of *P. aeruginosa*, but no decrease in viable count of *P. aeruginosa* was seen in the supernatants of different lactobacilli in 18 hrs. Research has demonstrated that this inhibitory activity of lactobacilli can be different in liquid medium compared to solid medium because of better diffusion of the substance secreted by lactobacillus (33).

In some studies, the probiotic activities of Lactobacillus administered vaginally have been evaluated in woman with urinary tract infection (34–37). In one of these studies, five females suffering from recurrent urinary tract infections were treated twice weekly with intra-vaginal and perineal implantation of *Lactobacillus casei* GR-1, and has been found that that *L. casei* GR-1 inhibited the growth of the coliforms bacteria (36). It has been showed that lactobacillus vaginal suppositories are safe and may be effective in reducing the recurrence of urinary tract infections (UTI) following three days antimicrobial therapy with norfloxacin or trimethoprim/sulfamethoxazole (TMP/SMX) in forty-one adult women with acute lower UTI (37).

Lactobacilli are able to inhibit the growth of *P. aeruginosa* by different mechanisms. These friendly bacteria could act as bio-therapeutic microorganisms and might be good candidates to overcome the growing challenge of nosocomial infections due to multi-drug resistant strains of *P. aeruginosa*.
